# Coating of chitosan on poly D,L-lactic-co-glycolic acid thymoquinone nanoparticles enhances the anti-tumor activity in triple-negative breast cancer

**DOI:** 10.3389/fchem.2023.1044953

**Published:** 2023-02-08

**Authors:** Jingrong Gao, Ankita Kumari, Xin-An Zeng, Siewyin Chan, Muhammad Adil Farooq, Mahafooj Alee, Shaheer Hasan Khan, Abdul Rahaman, Shan He, Xiong Xin, Tariq Mehmood

**Affiliations:** ^1^ School of Food and Pharmacy, Zhejiang Ocean University, Zhoushan, China; ^2^ School of Food Science and Engineering, South China University of Technology, Guangzhou, China; ^3^ Guangdong Provincial Key Laboratory of Intelligent Food Manufacturing, Foshan University, Foshan, China; ^4^ Overseas Expertise Introduction Center for Discipline Innovation of Food Nutrition and Human Health (111 Center), Guangzhou, China; ^5^ China-Singapore International Joint Research Institute, Guangzhou, China; ^6^ Institute of Materials Research and Engineering (IMRE), Agency for Science, Technology and Research (A*STAR), Singapore, Singapore; ^7^ Department of Food Science and Technology, Khwaja Fareed University of Engineering and Information Technology, Rahimyar Khan, Punjab, Pakistan; ^8^ Enzymology and nanotechnology laboratory, Interdisciplinary Biotechnology Unit, Aligarh Muslim University, Aligarh, India; ^9^ Institute for Nano Scale and Technology, College of Science and Engineering, Flinders University, Bedford Park, SA, Australia; ^10^ College of Engineering, Information, Technology & Environment, Charles Darwin University, Darwin, NT, Australia; ^11^ The Department of Anaesthesiology, The Second Affiliated Hospital of Guangzhou University of Chinese Medicine, Guangzhou, Guangdong, China

**Keywords:** thymoquinone, triple negative breast cancer, polymeric nanoparticles, polylactic acid, chitosan, hybrid nanoparticles

## Abstract

Breast cancer is the second most common cancer around the world. Triple-negative breast cancer (TNBC) is characterized by the absence of three receptors: progesterone, estrogen, and human epidermal growth factor-2 receptor (HER2). Various synthetic chemotherapies have gained attention but they caused unwanted side effects. Therefore, some secondary therapies are now becoming famous against this disease. For instance, natural compounds have been extensively researched against many diseases. However, enzymatic degradation and low solubility remain a major concern. To combat these issues, various nanoparticles have been synthesized and optimized from time to time, which increases its solubility and hence therapeutic potential of a particular drug increases. In this study, we have synthesized Poly D,L-lactic-co-glycolic acid (PLGA) loaded thymoquinone (TQ) nanoparticle (PLGA-TQ-NPs) and then coated them by chitosan (CS) (PLGA-CS-TQ-NPs), which was characterized by different methods. Size of non-coated NPs was 105 nm with PDI value of 0.3 and the size of coated NPs was 125 nm with PDI value of 0.4. Encapsulation efficiency (EE%) and Drug loading (DL%) was found to be 70.5 ± 2.33 and 3.38 for non-coated and 82.3 ± 3.11 and 2.66 for coated NPs respectively. We have also analysed their cell viability against MDA-MB-231 and SUM-149 TNBC cell lines. The resultant, nanoformulations exhibit anti-cancerous activity in a dose and time-dependent manner for MDA-MB-231 and SUM-149 cell lines with an IC_50_ value of (10.31 ± 1.15, 15.60 ± 1.25, 28.01 ± 1.24) and (23.54 ± 1.24, 22.37 ± 1.25, 35 ± 1.27) for TQ free, PLGA-TQ-NPs and PLGA-CS-TQ-NPs respectively. For the first time, we have developed a nanoformulations of PLGA loaded TQ coated with CS NPs (PLGA-CS-TQ-NPs) against TNBC which led to their enhanced anti-cancerous effects.

## Introduction

Breast cancer remains one of the deadliest diseases in women around the globe with 2,261,419 new deaths reported in 2020 (Sung et al., 2021). We can define triple-negative breast cancer (TNBC) as a major type of breast cancer in which estrogen (ER), progesterone (PR) and human epidermal growth factor receptor (HER-2) show negative expression profiles (Wolff et al., 2013). Comparatively, the survival rate is very less and the mortality rate is around 40% within the first 5 years of diagnosis (Dent et al., 2007).

Regarding the treatment options, chemotherapy is an effective treatment for TNBC in which various combination regimes comes out to be a positive approach like taxel/docetaxel + adriamycin + cyclophosphamide (TAC), adriamycin + cyclophosphamide (AC), cyclophosphamide + methotrexate + fluorouracil (CMF), docetaxel + cyclophosphamide (TC) (Yin et al., 2020). But these are synthetic chemo-drugs that impart heavy toxicity in addition to their effectiveness. So, to cope with this situation, the natural compound has been thoroughly searched and then extensively researched for its role against TNBC as they are very less toxic and cost-effective. Moreover, the main drawback with natural compounds is their low solubility which hampers their effectiveness. In the past few decades, nanotechnology has been an emerging field to address these issues, attracting many scientists to utilize them in medicine, especially in cancer treatment (Brigger et al., 2012; Khan et al., 2021). Among many approaches, drug delivery systems (DDS) remain one of the leading approaches for controlled drug delivery and toxicity-related issues (Khan et al., 2020; Adepu and Ramkrishna, 2021).

The vasculature of the tumor has been described as “leaky” due to pore size ranging from 0.2 to 1.2 µm (Yuan et al., 1995; Hobbs et al., 1998). This leaky environment of tumor vasculature promotes an effect known as the “enhanced permeation and retention (EPR) effect, which allows NPs to penetrate tumor vasculature, and thereby enhanced its therapeutic potential (Teicher, 2000; Sledge and Miller, 2003).

Poly-(lactic-co-glycolic acid) (PLGA) is the major polymeric NPs that is used as a drug delivery agent against various types of cancer owing to their biocompatibility and biodegradability (Dahhier et al., 2012; Varypataki et al., 2016). The property of burst release of their contents (Chen et al., 2016; Wang et al., 2018), and very less specific interaction with cells or proteins reduced the drug concentration on target cells (El-Hammadi et al., 2017; Wu et al., 2017). Therefore, they are being modified through various polymers like chitosan which enhances their cellular uptake and effectiveness (Chen et al., 2014; Taghavi et al., 2017). CS is a cationic polymer obtained by the deacetylation of chitin which is a natural polymer found in the cell walls of fungi and an important integral component of the exoskeleton of arthropods (Elieh-Ali-Komi and Hamblin, 2016). It is used as a drug delivery agent due to its amazing biocompatibility and biodegradability (Ali and Ahmed, 2018). Sesamol is an anti-cancer agent and its incorporation into cadmium sulphide (CdS) quantum dots (QDs) modified chitosan (CTS) greatly enhanced the drug loading activity and the anti-cancerous activity of sesamol (Abdelhamid et al., 2019). Chitosan also finds its place in using against cancer therapy, oral drug delivery, transdermal delivery *via* formation of chitosan hydrogels (Saeedi et al., 2022). Chitosan also finds its place in delivering oligonucleotides in cancer therapy. In this regard, chitosan-modified iron oxide magnetic nanoparticles was synthesized and checked for gene therapy (Dowaidar et al., 2018). Due to their positive nature, they can attach themselves to negatively charged membranes which results in their mucoadhesive property i.e., an important criterion for drug delivery systems. Hence, the coating of CS on PLGA NPs enhances its therapeutic potential.

Thymoquinone (TQ), obtained from seeds of *Nigella sativa* or black seeds possess many benefits in addition to their anti-cancerous property (Randhawa and Al-Ghamdi, 2002). TQ proved to be an anti-cancerous agent against TNBC through various mechanisms and modulation of the tumor micro-environment (Adinew et al., 2021). Various nanoparticles of TQ has been synthesized to encapsulate it and increase its solubility and effectiveness. e.g., TQ was loaded in lipid nanostructured lipid carrier strikingly enhanced the anticancer activity in 4T1 tumor bearing mice in breast cancer mode (Ong et al., 2018). Similarly, TQ also incorporated in solid lipid nanoparticles (SLNs) and demonstrated antidepressant like property in rats (Alam et al., 2020). TQ also inhibits cervical cancer which was proved by loaded TQ in mesoporous silica nanoparticles which results inhibition by retarding of cell invasion and ROS mediated apoptosis (Goel and Mishra, 2019). So, we have summarized the previous works done using TQ as a therapeutic agent in table. But, none of the literature found that optimized TQ loaded in PLGA NPs and coated with CS, treated with a Triple-negative breast cancer cell line. Activity of TQ nanoformulations on different diseases summarized in Table 1.

**TABLE 1:** Illustrates important nanoformulations of TQ and their roles.

TQ nanoformulations	Activity	References
TQ- lipid nanostructured nanocarrier	Enhanced activity in 4T1 cancer bearing breast cancer	Ong et al. (2018)
TQ- solid lipid nanoparticles	Antidepressant like property in rats	Alam et al. (2020)
TQ-mesoporous silica nanoparticles	Enhanced anti-cervical cancer property	Goel and Mishra, (2019)

In the view of above updates, we have synthesized PLGA-CS-TQ-NPs and then evaluated their therapeutic potential against TNBC. This study will be the first and novel study to find out the effective role of TQ against deadly TNBC.

## Materials and methods

### Materials

Thymoquinone, PLGA, chitosan and polyvinyl alcohol (PVA), sodium hydroxide were purchased from sigma Aldrich (St. Louis, Missouri, United states), while dimethyl sulphoxide (DMSO), acetonitrile were purchased from Shanghai Fuchen Chemical Industry Limited Company, Qingpu District, Shanghai, China. 3-(4,5-dimethylthiazol-2-yl)-2,5diphenyl-2-H-tetrazolium bromide bromide, 4’,6-diamidino-2-phenylindole (DAPI) (MTT) were procured from Shanghai Lingfeng Chemical Reagent Co., Ltd. China. All other reagents used were purchased from Tianjin Kermel Chemical Reagent Co. (China). SUM 169 and MBD MB-231 triple-negative breast cancer cell lines were obtained from ATCC.

### Methods

#### Synthesis of TQ-loaded PLGA nanoparticles

PLGA CS modified NPs were synthesized by the nanoprecipitation method using the protocol optimized by Lu et al. with little modification. First of all, we synthesized PLGA-TQ-NPs (non-coated NPs) and then we coated CS on these synthesized NPs. Briefly, PLGA (100 mg) and thymoquinone (50 mg) were subsequently mixed with acetone (10 mL) to form the organic first phase. Polyvinyl alcohol (PVA) (50 mg) was slowly poured into the deionized water (200 mL) to form the second phase, the aqueous phase. Following this, both phases at moderate flow rates were pumped into the RPB reactor for the complete mixing of two solutions to form PLGA-NPs. On these NPs coating of CS (30 mg) was done by dissolving CS in 0.5% acetic acid aqueous solution to form (PLGA-CS-TQ-NPs) on a magnetic stirrer at a speed of 1,000 r.p.m (Lu et al., 2019). The non-loaded drug was separated by high-speed centrifugation (15,000 r/min, 15 min). TQ-loaded NPs remained in the pellet after the supernatant was discarded and the nanoparticles were again re-suspended in distilled water after mixing it with 5% glucose for the lyophilization step for a few hours (vacuum freeze-drying machine, Lycodel, China). Further, the characterization and *in vitro* studies were performed on these re-suspended nanoparticles by following different protocols. Void NPs were also synthesized using the same protocol as above without using TQ.

### Characterization of nanoparticles

#### Hydrodynamic radii, zeta potential, and surface morphology of NPs

The size of NPs was measured using a zeta sizer instrument (Malvern ZS nano) by utilizing the phenomenon of dynamic light scattering. Zeta potential was also measured from the same instrument by just changing the program. The sample’s readings were taken thrice after diluting it with double distilled water. The size of NPs was captured by Transmission electron microscopy (TEM, model number-0000, JEOL, Tokyo, Japan) using uranyl phosphate as a staining agent. Shape and Morphology of nanoparticles was captured with a scanning electron microscope (SEM) by casting a drop of sample onto the coverslip and then it was dried for at least 1 day. This dried sample was then coated with gold using a gold coating sputter (Astete and Sabliov, 2006).

#### Drug loading and encapsulation efficiency

TQ was quantified by using UV-Vis spectroscopy at a lambda max peak at 254 nm which shows how much drug is entrapped within nanoparticles. For this lyophilized NPs were treated with triton-X-100 (1%) to release the drug from NPs which was quantified spectrophotometrically. For this, standard curve for TQ was plotted by taking a fixed concentration and then increasing that concentration which was plotted on a graph to find the value of the amount of drug loaded in NPs. So, we can say that (EE) is the percentage of the amount of drug loaded in NPs and the weight of the drug initially used during the preparation of NPs. The mathematical formula for calculating EE is as follows:

Encapsulation efficiency (%) = Total drug (mg)—Free drug (mg) X100 Total drug (mg).

And drug loading percentage may be defined as the amount of drug is present in nanoparticles divided by total weight of nanoparticles including drug. The mathematical Formula 1 for calculating LC is as follows:

Drug loading (%) = Weight of drug in NPs (mg) X100%

$$Weight\;of\;NPs\;\left(mg\right)$$ (1)

#### *In vitro* release kinetics

Around 50 mg TQ-loaded NPs were redispersed in 5 mL double distilled water and poured into a dialysis membrane bag having a molecular cut-off of 12 kDa. This bag was kept in a phosphate buffer having pH 7.4 and temperature 37°C to understand the rate of release at physiological pH conditions with continuous stirring at room temperature. After predefined intervals, 2.5 mL of water is removed from the beaker and the same amount was filled into the beaker. Same experiment was repeated for cancer microenvironment pH which was around 5.5. The amount of TQ was measured spectrophotometer at 254 nm wavelength using a calibration curve. Experiments were replicated for 20 days and the release content of TQ was plotted against the number of days in which experiments were performed.

Also, we analysed the *in vitro* drug release data through various kinetic models to describe the release kinetics of nanoparticles. Here, the zero order rate Eq. 2 explains the systems in which the rate of drug release does not depend on its concentration (Dash et al., 2010). Also, the first order rate kinetics were given using Eq. 3 which explains the release from the system in where rate of release of drug is concentration dependent (Costa and Lobo, 2001). Furthermore, Higuchi (Higuchi, 1963) also described the release pattern of drugs from insoluble matrix as a square root of time dependent process which was based on Fickian diffusion as given in Eq. 4. Finally, another model which was described by Korsmeyer et al. (1983) which derived a simple mathematical formula which explained the drug release from a polymeric system as given by Eq. 5.

$$C=k_{o}t$$ (2)

Where, C is the concentration of drug at time t, t is the time and k_o_ is zero-order rate constant expressed in units of concentration/time.

$$\mathrm{Log}\;C_{O\;–}\mathrm{Log}\;C=k_{1t}\;/\;2.303$$ (3)

Where, C_O_ is the initial concentration of drug and k_1_ is the first order rate constant.

$$C=K_{H}\;t^{1/2}$$ (4)

Where, K_H_ is the constant reflecting the variable of the system.

$$M_{t}\;/\;M_{\infty }=K_{{KP}^{tn}}$$ (5)

Where, M_t_/M_∞_ is the fraction of drug released at time t, K_KP_ is the rate constant and n is the release exponent.

#### Cellular uptake studies

How much NP accumulates into TNBC cells is known as the cellular uptake of NPs was examined by observing rhodamine B-filled nanoparticles into SUM-149 cells through a confocal laser scanning microscope (CLSM). TNBC cell lines were grown to 60%–70% population on sterile coverslips for further procedures. Furthermore, cells were treated with different NPs for around 3 h which was followed by PBS buffer washing to remove non-loaded NPs. After this, DAPI fluorescent was used to stain the nucleus for further analysis. Uptake studies of our nano-formulation were then visually observed under CLSM on (CLSM, D-Eclipse C1, Nikon).

#### MTT assay

The cytotoxicity of free TQ, PLGA-TQ-NPs, and PLGA-CS-TQ-NPs was examined using the MTT on MDA-MBA 231 and SUM-149 cell lines. Around 5 × 103 cells were plated in 96 well plates and cultured for 24 h at 37°C in 5% CO_2_ in Dulbecco’s modified eagle medium (DMEM) which also contains 10% fetal bovine serum. After 24 h, cells were treated with different concentrations of free TQ, loaded TQ NPs, DMSO, or blank NPs for around 72 h. About 50 µL of MTT was added following drug and NPs treatment and incubated for another 3 h. Later, MTT was removed and 50 μL of ethanol and 150 μL of isopropyl alcohol (1:2) solution were added into it to solubilize the formed formazan crystals which were analyzed in a micro plate spectrophotometer at a wavelength of 570 nm to calculate half maximum inhibitory drug concentration (IC50) by using Graph Pad Prism software. The cell viability was calculated using the below Eq. 6, where the Abs sample is the absorbance of treated cells and Abs control is the absorbance of untreated cells (Liu et al., 2019).

$$Cell\;viability=\frac{Abs\;sample}{Abs\;Control}$$ (6)

#### Statistical analysis

The readings were taken in triplicate and then their differences in average were compared by simple analysis of variance (one-way ANOVA, GraphPad Instat 3) or independent sample *t*-test (Origin 6.1 USA). The significance of the difference was determined at the 95% confidence limit (*α* = 0.05).

## Results and discussion

### Hydrodynamic radii, polydispersity index, zeta potential, and surface morphology of NPs

**FIGURE 1:**
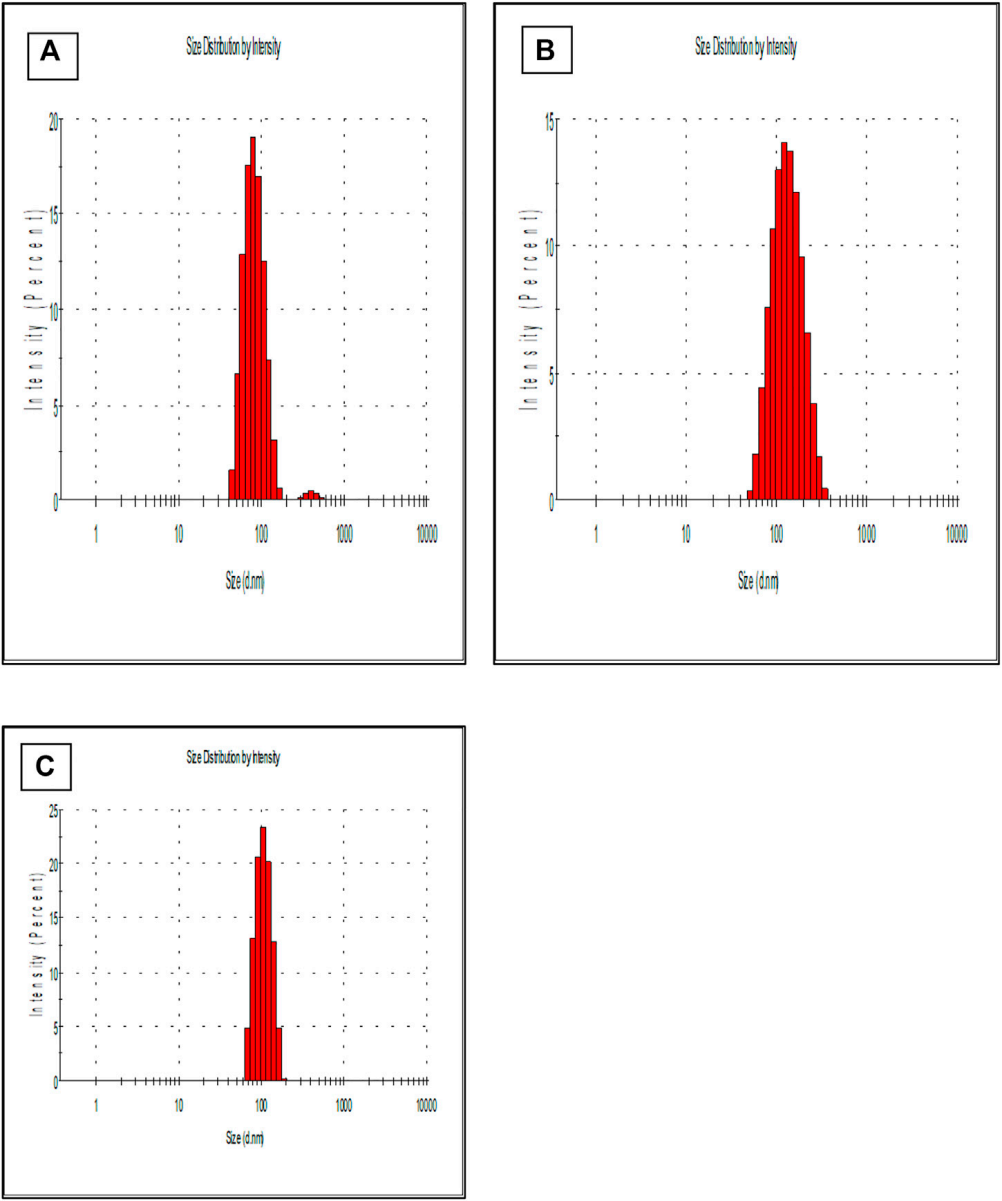
Shows the size of **(A)** PLGA-Void-NPs, **(B)** PLGA-TQ-NPs, and **(C)** PLGA-CS-TQ-NPs using the principle of dynamic light scattering.

**FIGURE 2:**
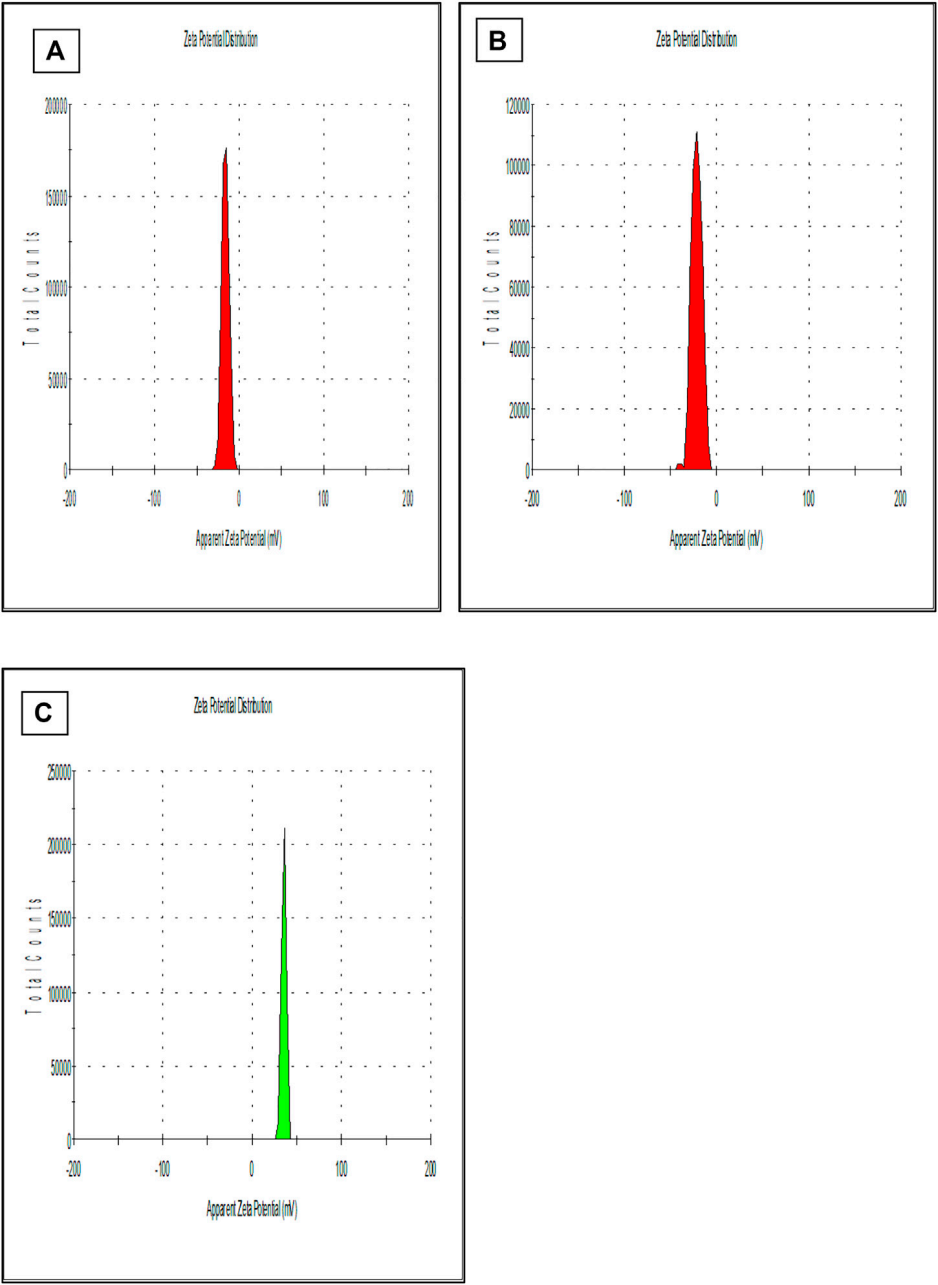
Shows the zeta potential of **(A)** PLGA-Void-NPs, **(B)** PLGA-TQ-NPs, and **(C)** PLGA-CS-TQ-NPs.

Previous studies reported that the nano-particles smaller than 10 nm can be excreted from the kidneys, while larger particles (˃300 nm) can be eliminated from the systemic circulation when identified by the reticuloendothelial system (RES) (Fox et al., 2009; Kobayashi et al., 2014). Figure 1 is showing a data from the zeta sizer which showed that nanoparticles had a size range of 80 nm–150 nm which increases upon coating with CS. Further, polydispersity index (PDI) values of coated and non-coated NPs range from 0.2–0.3 also indicating a stable system. It is reported that lower the value of PDI, higher probability of monodisperse system is found (Rao et al., 2011; Mudalige et al., 2019). Similarly, Othman et al. TQ and Ascorbic acid in chitosan also showed particle PDI value of about 3.8 (Othman et al., 2020). Furthermore, non-coated PLGA NPs showed a negative zeta potential (−21.7 ± 2.23) due to the presence of carboxyl groups at the end of the PLGA chain. PLGA-CS-NPs exhibit a positive zeta potential (+35.6 ± 3.22) (Figure 2), due to the presence of amino groups at the surface of CS This transition of potential from negative to positive is a clear indication of the coating of CS on PLGA NPs (Xiao et al., 2016). It may illustrate that this positive potential interact with the negative charge of plasma membrane due to which it gets attached with it thereby enhancing the cellular uptake of tumor cells (Abouelmagd et al., 2015; Shariatinia, 2019). The major characteristics of NPs formed are shown in Table 2.

**TABLE 2:** Major characteristics of NPs formed.

Nanoparticles	Size (d. nm) Av	Polydispersity index	Zeta potential (mV)
PLGA-NPs	94.99 ± 1.76	0.2	−16.3 ± 2.45
PLGA-TQ-NPs	105 ± 2.66	0.3	−21.7 ± 2.23
PLGA-CS-TQ-NPs @ 30% CS.	122.3 ± 2.54	0.3	+35.6 ± 3.22

**FIGURE 3:**
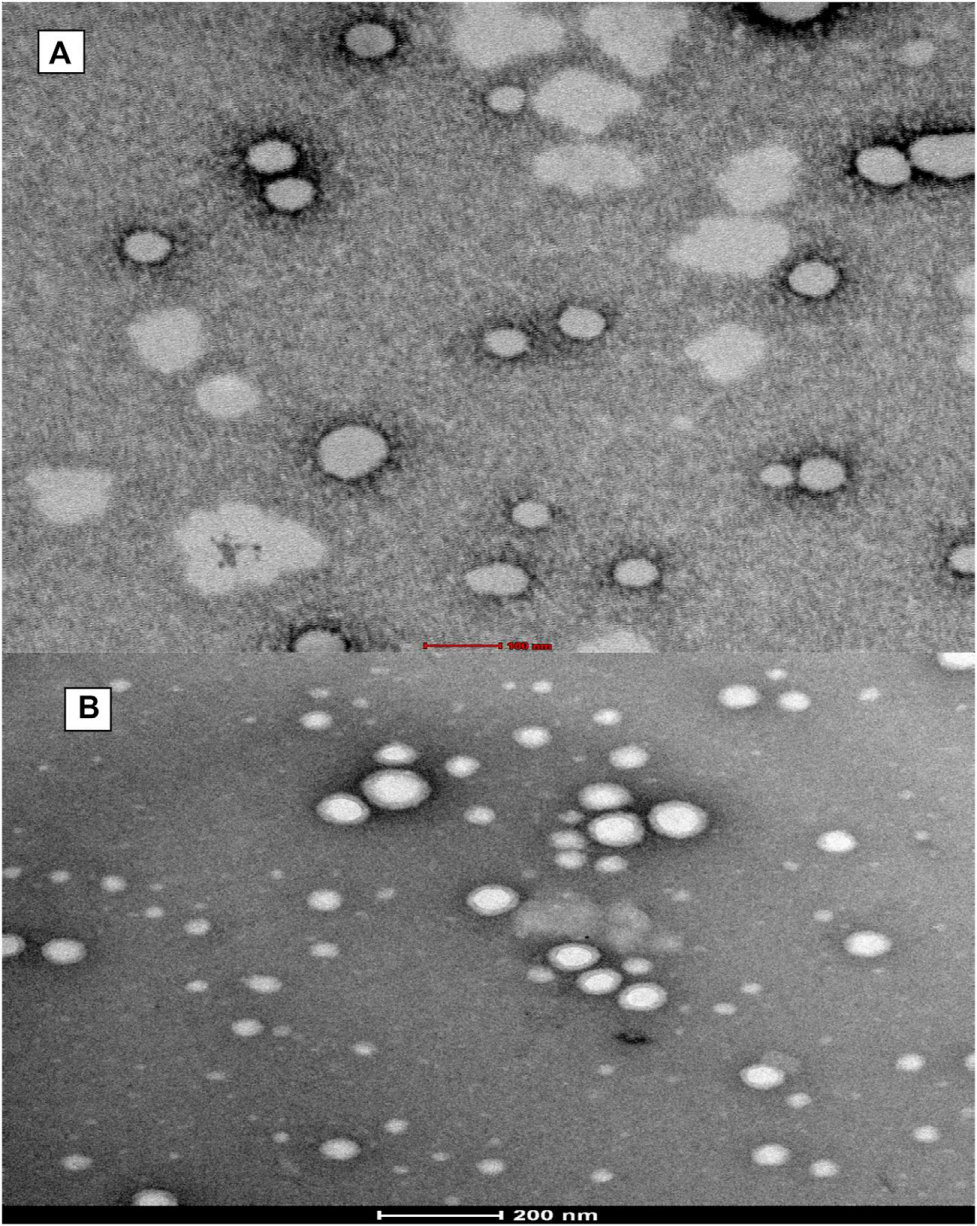
Shows transmission electron microscopy (TEM) of formed **(A)** PLGA-Void-NPs and **(B)** chitosan-modified PLGA nanoparticles (PLGA-CS-TQ-NPs).

HR-TEM (Figure 3) images confirmed the spherical shape of NPs and undeviating size distribution. Moreover, the diameter of NPs recorded from HR-TEM is smaller (100–150 nm) than recorded by DLS due to the principle that the former measured the hydrodynamic radii in which coating of water is also present while it is absent in the latter (dried HR-TEM) characterization samples (Hassellöv et al., 2008).

**FIGURE 4:**
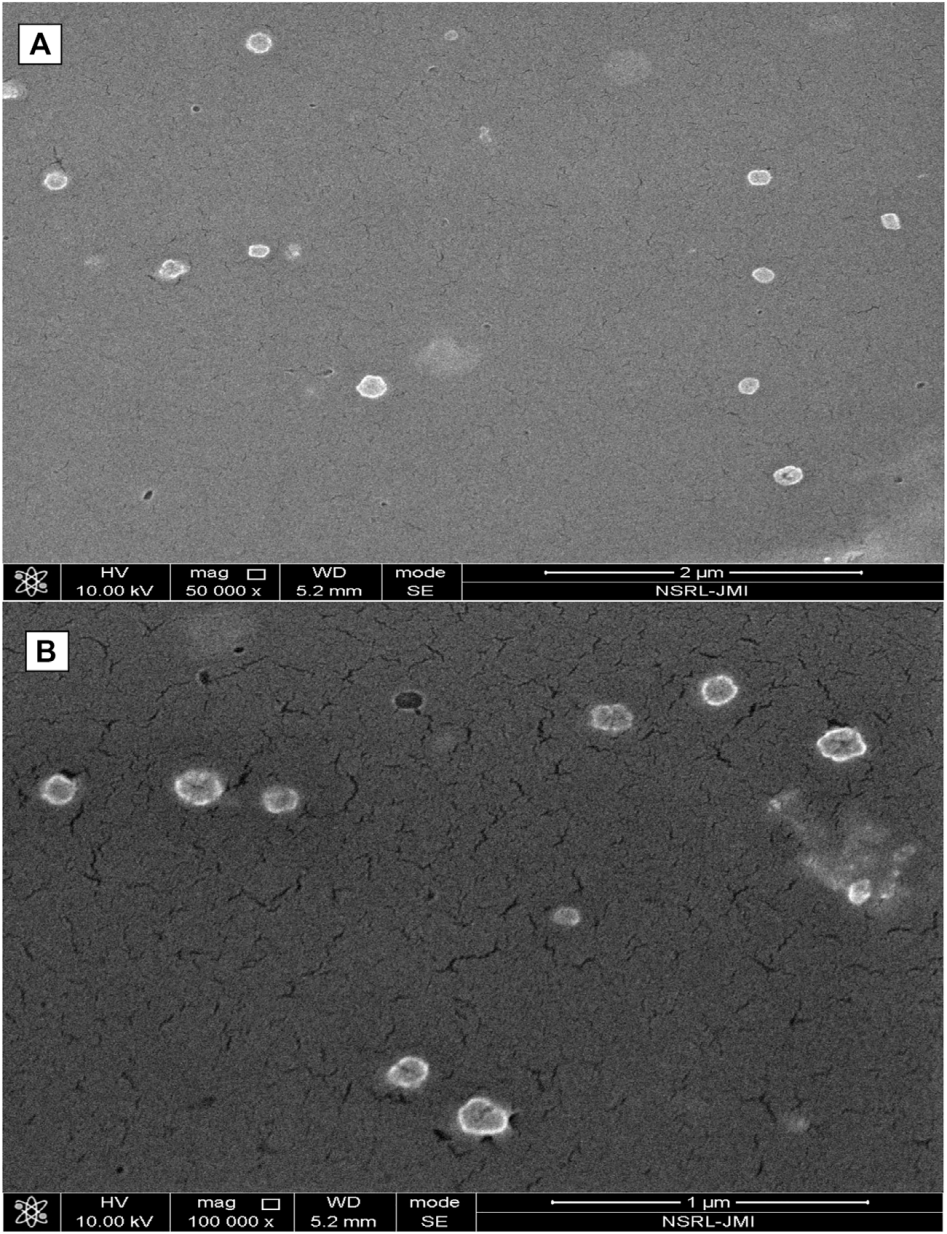
Shows field emission scanning electron microscopy (Fe-SEM) of formed **(A)** PLGA-Void-NPs and **(B)** chitosan-modified PLGA nanoparticles (PLGA-CS-TQ-NPs).

Fe-SEM (Figure 4) also signified the shape of formed nanoparticles which showed spherical particles with a size range of 100 nm–150 nm. At some places in the photograph, a few nanoparticles collide with each other to form elongated aggregated particles.

### Encapsulation efficiency of formed nanoparticles

EE of formed PLGA-TQ-NPs was found to be 70.5%. Moreover, the value of EE for CS-modified PLGA-CS-TQ-NPs was 82.3%. TQ is a hydrophobic drug that was encapsulated in PLGA NPs to avoid contact with an aqueous environment. Drug and PLGA dissolve in the organic solvent while the emulsifier is dissolved in water. When these two contacts each other, solvent displacement occur at the interface of two phases. Then, TQ and PLGA formed into NPs at the interface (Fessi et al., 1989; Almoustafa et al., 2017; Rivas et al., 2017). Some molecules of drugs leaked from the nanoparticles resulting in their low EE, while CS decreased the amount of drug coming out from PLGA-NPs (Lu et al., 2019). Hence, coating of CS around NPs was a good strategy to increase the EE of TQ besides several other benefits. EE % and DL % is summarized in Table 3.

**TABLE 3:** Represents the type of NP and their encapsulation efficiency (EE%) and Drug loading (DL%).

Nanoparticle type	Encapsulation efficiency %	Drug loading (DL)
PLGA-TQ-NPs	70.5 ± 2.33	3.38
PLGA-CS-TQ-NPs	82.3 ± 3.11	2.66

### *In vitro* release kinetics

**FIGURE 5:**
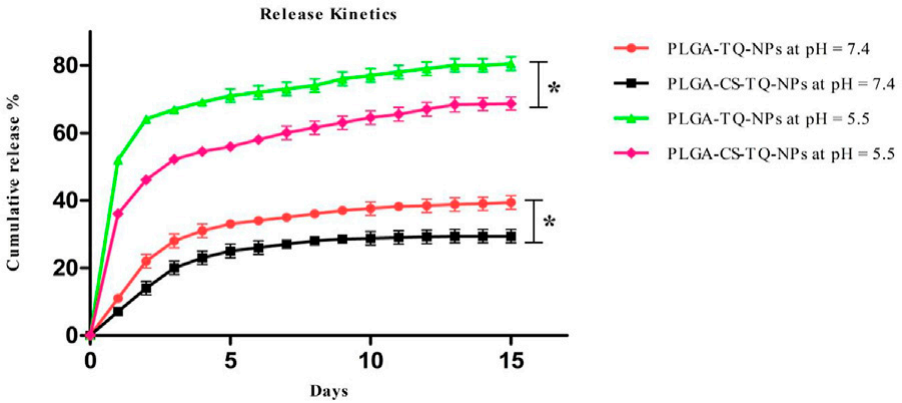
Illustrates the *in-vitro* release profile of PLGA-TQ-NPs and PLGA-CS-TQ-NPs at pH = 7.4 and pH = 5.5.

How much of the drug has been released at different pH is determined by *in vitro* release kinetics. NPs release their drug slowly and in a sustained manner at 7.4 which is our physiological pH. The cumulative release value of PLGA-TQ-NPs and PLGA-CS-TQ-NPs in 15 days at physiological pH was found to be 39.4 ± 2.1% and 29.4 ± 2.01% respectively. The initial burst drug release was most likely due to the release of TQ that loosely bound to the surface of the NPs. Whereas the latter sustained release showed a slow release which might be due to the TQ release from the core of PLGA nanoparticles because of swelling and hydration of NPs matrix. Furthermore, the coating promoted the burst release of drugs from the NPs core by protecting the leaky behaviour pattern of PLGA NPs. Due to this effect drug released at a slower rate in case of CS coated nanoparticles than CS non-coated nanoparticles. Pattern of release kinetics at acidic pH which is about 80.5 ± 2.0 and 68.7 ± 1.9 for PLGA-TQ-NPs and PLGA-CS-TQ-NPs respectively was higher and rapid due to the fact that in acidic environment PLGA was degraded into its monomer which results into faster release from the PLGA NPs. The result was same as obtained in previous studies (Lu et al., 2019). These results showed that TQ was effectively entrapped within the PLGA NPs matrix and released their contents effectively, slowly, and in a sustained manner. The released kinetics at both the pH has shown in Figure 5.

**FIGURE 6:**
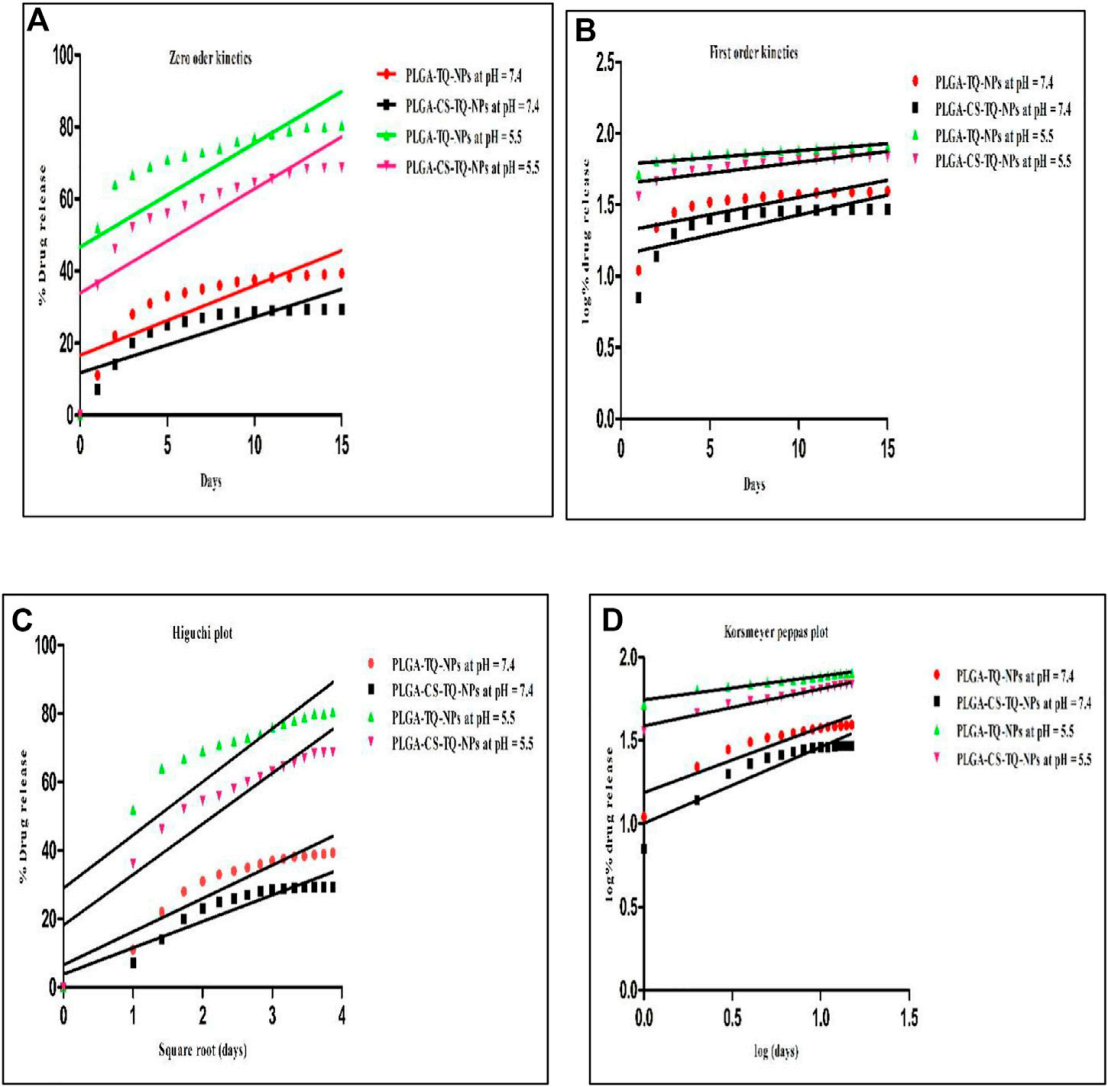
Showed different models of release kinetics **(A)** zero order kinetics model **(B)** first order kinetics model **(C)** Higuchi model **(D)** Korsmeyer Peppas model plot.

Kinetic study plots were plotted for different models: cumulative % drug release vs. time (zero order model), log % drug release vs. time (first order kinetics model), cumulative % drug release vs. square root of time (Higuchi model), log cumulative % drug release vs. log time (Korsmeyer-Peppas model). All the plots of different models were shown in Figure 6 And the results were summarized in Table 4, where *R* ^2^ is the correlation value, which indicates best fit (highest correlation value). Different *R* ^2^ values indicates how well the data fit the regression model which is also known as goodness of fit. In Table 4, we can see the *R* ^2^ value is highest for PLGA-CS-TQ-NPs at pH = 5.5, which is obviously tumor microenvironment. Here we can have concluded that the nanoparticles followed Korsmeyer-Peppas model with a correlation value 9.555, which was highest among all the other groups and was significant with previous reports (Bohrey et al., 2016).

**TABLE 4:** Represents the *R* ^2^ (correlation value) values obtained from different models.

Types of nanoparticles and their *R* ^2^	Zero order	First order	Higuchi model	Korsmeyer peppas model
PLGA-TQ-NPs (pH = 5.5)	0.4841	0.7339	0.7298	0.9170
PLGA-CS-TQ-NPs (pH = 5.5)	0.6274	0.7714	0.8514	0.9558
PLGA-TQ-NPs (pH = 7.4)	0.6626	0.5429	0.8668	0.8278
PLGA-CS-TQ-NPs (pH = 7.4)	0.6624	0.5286	0.8537	0.8199

### Cellular uptake

Cellular uptake of PLGA-CS-TQ-NPs was visualized by CLSM which showed intense red color florescent cytoplasm in nanoparticle-treated SUM-149 cells. Here, the red color indicated that the NPs have been reached in these cancer-affected cells’ cytoplasm. As we have used rhodamine B dye for cellular uptake study, hence this dye emits red color when present in the cytoplasm of cells. In other words, rhodamine binds to the cytoplasm of cells and emits the red color fluorescent signal. A similar observation was seen in the case of DAPI staining which stains the nucleus of cells and gives blue color intensity. Our results, has been supported by the use of DAPI stain that the NPs reached up to the nucleus of SUM-149 cells. In a nutshell, we can say that the formed NPs efficiently internalized in SUM-149 TNBC cell lines and thus proven good drug delivery agent for cancer therapy. It has been illustrated in Figure 6.

### Cell proliferation assay (MTT assay)

**FIGURE 7:**
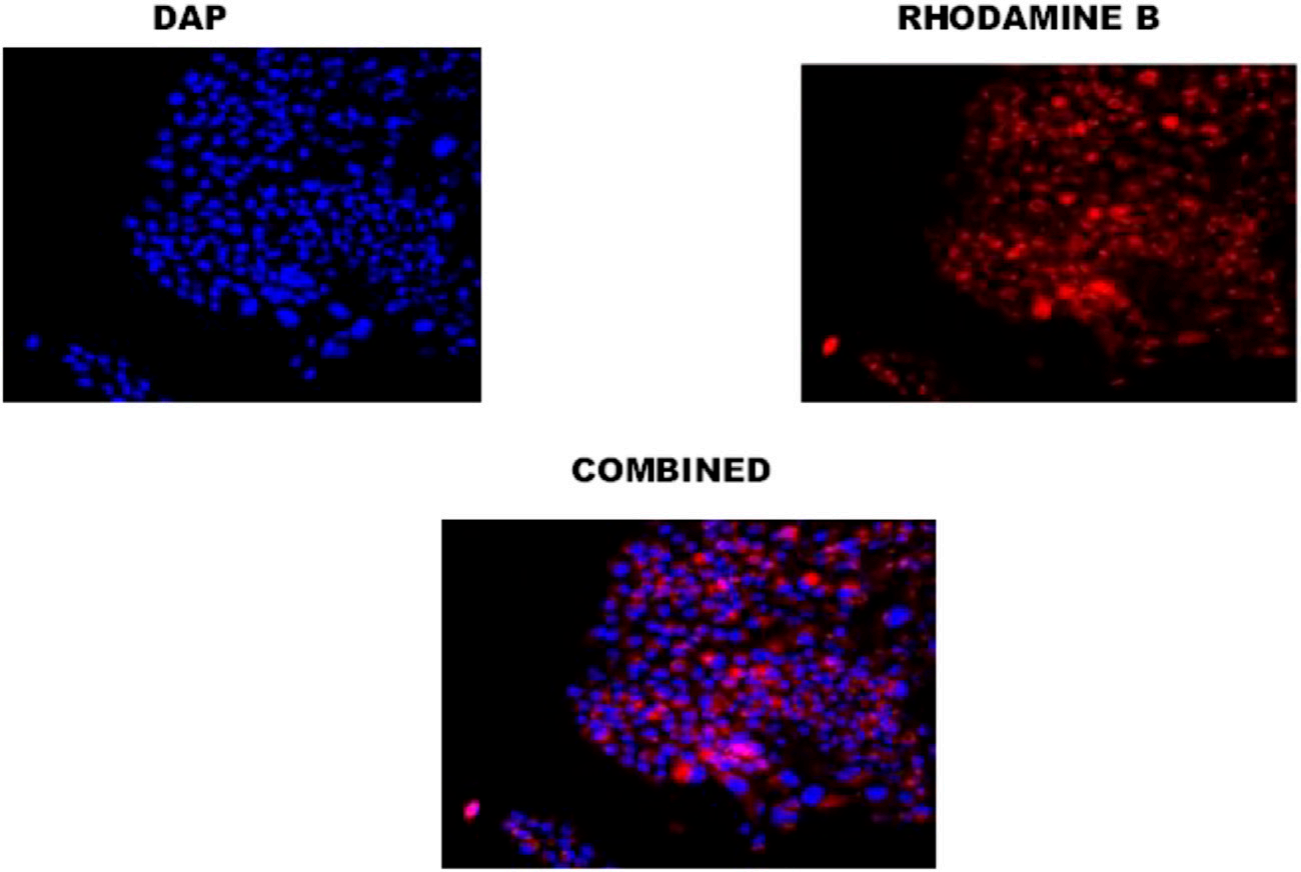
Confocal image of cellular uptake studies of TNBC cells after incubating with PLGA-TQ nanoparticles.

Cytotoxicity of synthesized NPs was assessed by using two triple-negative breast cancer cell lines: SUM-149 and MDA-MB 231, through cell proliferation assay (MTT assay) that were treated for 72 h. The graph shown in Figure 7 represents the percentage of cell viability versus the concentration of NPs and drugs used. It was found that both formulations showed concentration-dependent cell cytotoxicity against the TNBC cell line. IC_50_ values of free TQ, PLGA-TQ-NPs and PLGA-CS-TQ-NPs was found to be (10.31 ± 1.15, 15.60 ± 1.25, 28.01 ± 1.24) and (23.54 ± 1.24, 22.37 ± 1.25, 35 ± 1.27) against MDA-MB-231 and SUM-149 cell lines respectively.

**FIGURE 8:**
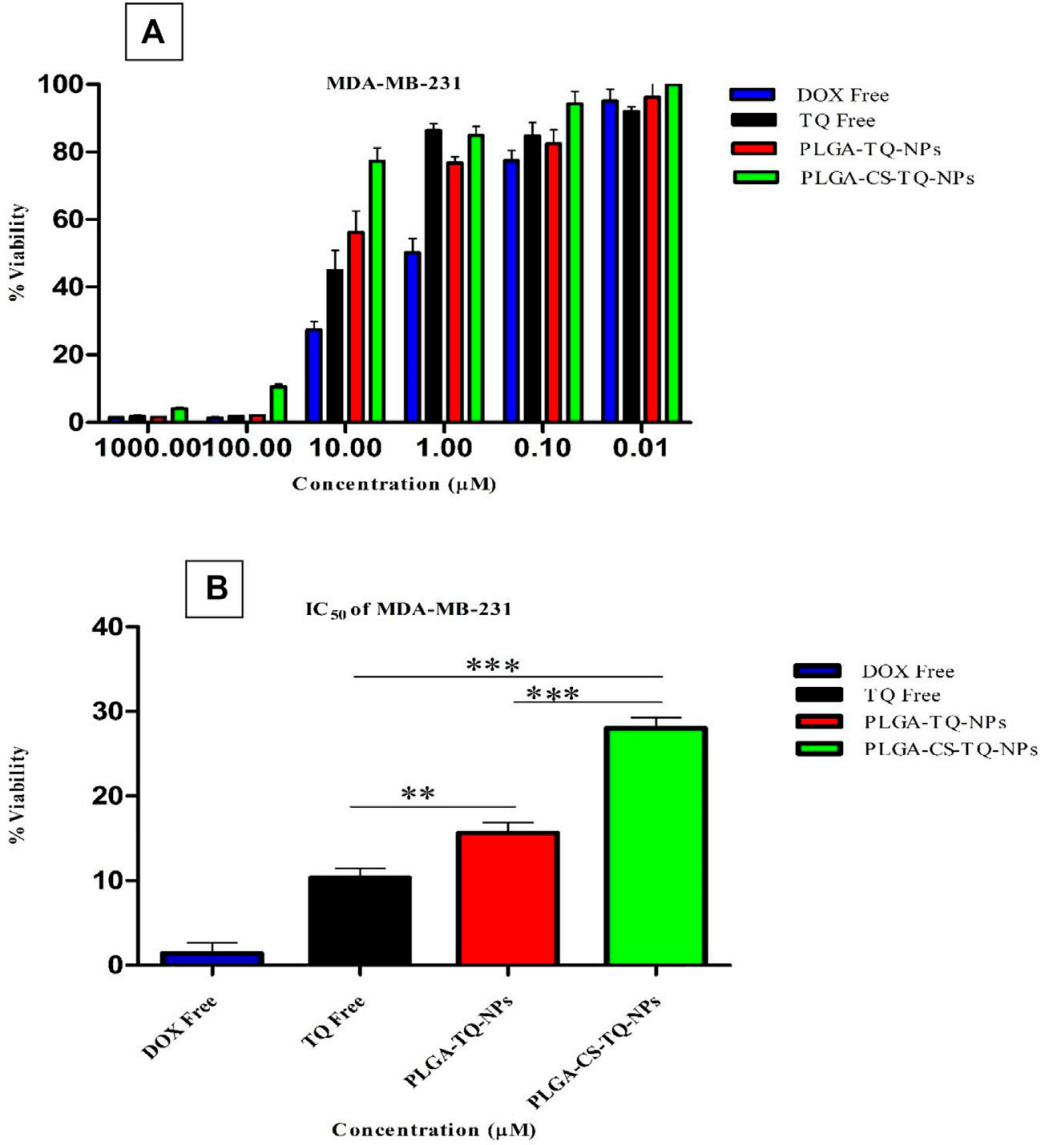
**(A)** Depicts the cell viability of TQ and its nanoformulations on MDA-MB-231 cells. **(B)** Comparison of inhibition at IC_50_ value by TQ and its nanoformulations.

**FIGURE 9:**
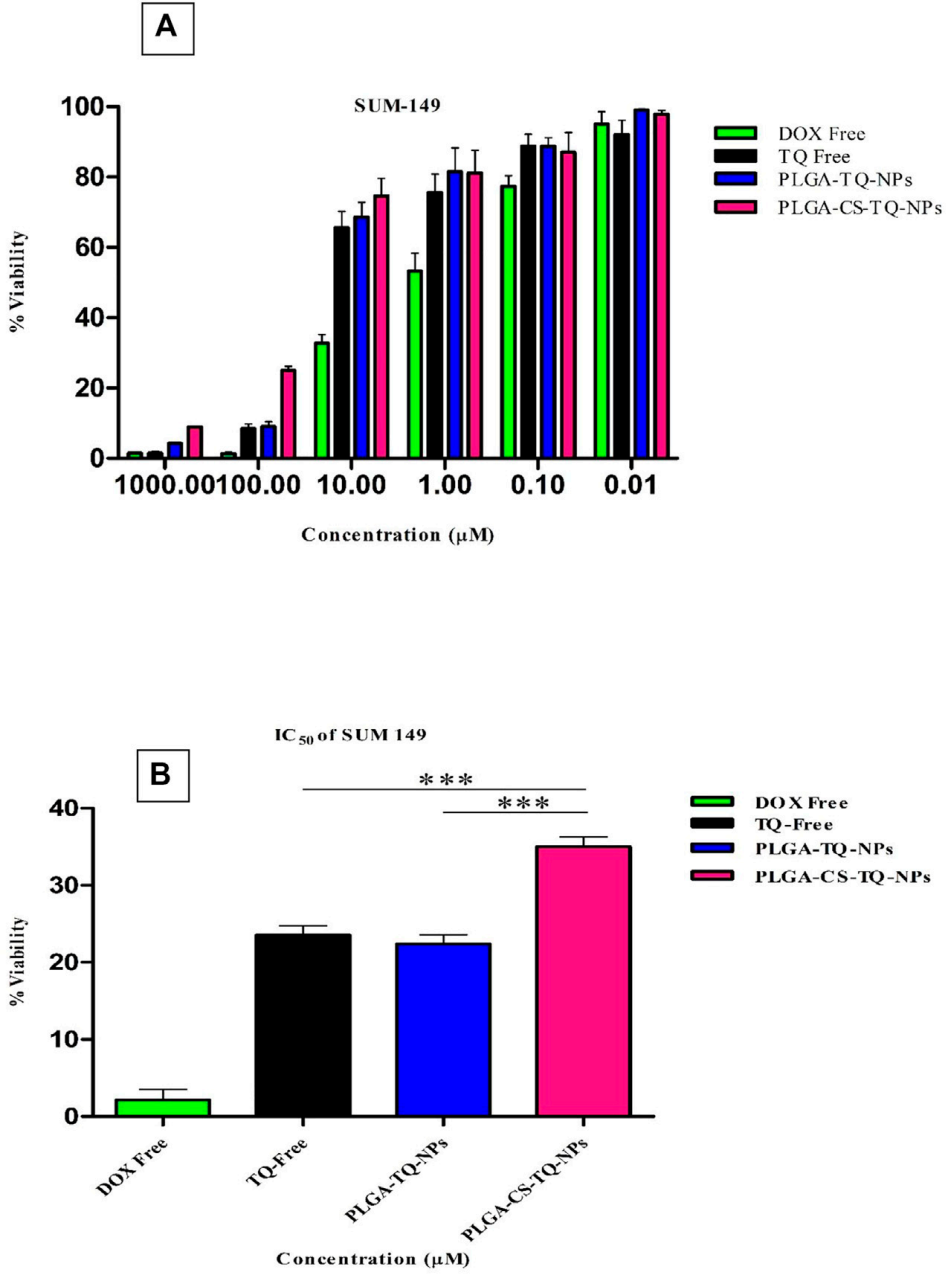
**(A)** Depicts the cell viability of TQ and its nanoformulations on SUM 149 cells. **(B)** Comparison of inhibition at IC_50_ value by TQ and its nanoformulations.

As mentioned in the Table 5, and in Figures 8, 9 that both the formulations had a lower cytotoxicity than free TQ which was in consistence with the results provided by (Ibrahim WN) (Ibrahim and Rosli, 2020). This is obvious because drug released from the nanoformulations slower and hence can act on cells at a slower pace than the free drug which is freely available to these cells. Cell takes drug by endocytosis results in increasing their concentration (Cartiera et al., 2009). Furthermore, due to additional controlled released behaviour of chitosan on PLGA (Lu et al., 2019), nanoparticles released their cargo at a slower rate than PLGA nanoparticles, which further decreased their *in vitro* cytotoxic potential. Moreover, nanoparticles give advantages in vivo systems than *in vitro* systems by increasing the bioavailability of drugs due to their prolonged circulation, enhancing aqueous solubility and enhanced targeting of nanoparticles towards cancer tissue (Kalyane et al., 2019).

**TABLE 5:** Represents the IC_50_ of PLGA NPs with and without CS coating against MDA-MB 231 and SUM 143 TNBC cell line treated for 72 h.

Formulation	IC_50_ (MDA MB 231 cell line)	IC_50_ (SUM-149 cell line)
DOX Free	1.369 ± 1.29	2.134 ± 1.384
TQ Free	10.31 ± 1.15	23.54 ± 1.24
PLGA-TQ-NPs	15.61 ± 1.25	22.37 ± 1.26
PLGA-CS-TQ-NPs	28.01 ± 1.24	35.00 ± 1.26

## Conclusion

In this work, we have synthesized chitosan-coated PLGA-TQ-NPs (PLGA-CS-TQ-NPs). The size of the nanoparticles formed was around 105 and 125 nm which was also verified by TEM images. This small size is a good idea for the EPR effect due to which NPs can be easily passed to the cells and hence increases the bioavailability of drugs to the tumor site. Zeta potential showed a negative potential of PLGA-loaded TQ, in which the charge was changed into a positive value when coated with chitosan. This coating prevents the burst release of drugs from the formed nanoparticles, which is evident from the released profiles at both the pH (normal and cancer tissue microenvironment). Furthermore, it interacts with the negative membrane of plasma membrane which results in adherence and immobilization of NPs around the tumor tissue. Both these effects prevent unwanted drug transfer and therefore lowered toxicity associated with several drugs and increased cytotoxic effects. Although TQ is known to have very less toxicity because it is natural in origin but having less water solubility hampers its efficacy which would increase by encapsulating it in nano-formulations. PLGA is a good choice of nano-formulation, however, its burst release sometimes hampers its normal drug delivery, thence coating it with certain polymers like CS is a good choice for lowering its burst release which increases its therapeutic efficacy. Due to controlled release behaviour, our system releases their cargo in a better way in *vivo* systems which are an essential feature of drug delivery system.

## Data Availability

The raw data supporting the conclusion of this article will be made available by the authors, without undue reservation.
